# A review of bat hibernacula across the western United States: Implications for white-nose syndrome surveillance and management

**DOI:** 10.1371/journal.pone.0205647

**Published:** 2018-10-31

**Authors:** Theodore J. Weller, Thomas J. Rodhouse, Daniel J. Neubaum, Patricia C. Ormsbee, Rita D. Dixon, Diana L. Popp, Jason A. Williams, Scott D. Osborn, Bruce W. Rogers, Laura O. Beard, Angela M. McIntire, Kimberly A. Hersey, Abigail Tobin, Nichole L. Bjornlie, Jennifer Foote, Dan A. Bachen, Bryce A. Maxell, Michael L. Morrison, Shawn C. Thomas, George V. Oliver, Kirk W. Navo

**Affiliations:** 1 USDA Forest Service, Pacific Southwest Research Station, Arcata, California, United States of America; 2 National Park Service Upper Columbia Basin Network, Bend, Oregon, United States of America; 3 Colorado Parks and Wildlife, Terrestrial Section, Grand Junction, Colorado, United States of America; 4 USDA Forest Service, Pacific Northwest Region, Eugene, Oregon, United States of America; 5 Idaho Department of Fish and Game, Boise, Idaho, United States of America; 6 Oregon State University – Cascades Campus, Human & Ecosystem Resiliency & Sustainability Lab, Bend, Oregon, United States of America; 7 Nevada Department of Wildlife, Ely, Nevada, United States of America; 8 California Department of Fish and Wildlife, Nongame Wildlife Program, Wildlife Branch, Sacramento, California, United States of America; 9 Western Cave Conservancy, Newcastle, California, United States of America; 10 Wyoming Game and Fish Department, Nongame Program, Lander, Wyoming, United States of America; 11 Arizona Game and Fish Department, Phoenix, Arizona, United States of America; 12 Utah Division of Wildlife Resources, Salt Lake City, Utah, United States of America; 13 Washington Department of Fish and Wildlife, Olympia, Washington, United States of America; 14 National Speleological Society, Santa Fe, New Mexico, United States of America; 15 Montana Natural Heritage Program, Helena, Montana, United States of America; 16 Texas A&M University, Department of Wildlife and Fisheries Sciences, College Station, Texas, United States of America; 17 Bat Conservation International, Subterranean Program, Olympia, Washington, United States of America; 18 Colorado Division of Wildlife, Monte Vista, Colorado, United States of America; CSIRO, AUSTRALIA

## Abstract

Efforts to conserve bats in the western United States have long been impeded by a lack of information on their winter whereabouts, particularly bats in the genus *Myotis*. The recent arrival of white-nose syndrome in western North America has increased the urgency to characterize winter roost habitats in this region. We compiled 4,549 winter bat survey records from 2,888 unique structures across 11 western states. *Myotis* bats were reported from 18.5% of structures with 95% of aggregations composed of ≤10 individuals. Only 11 structures contained ≥100 *Myotis* individuals and 6 contained ≥500 individuals. Townsend’s big-eared bat (*Corynorhinus townsendii*) were reported from 38% of structures, with 72% of aggregations composed of ≤10 individuals. Aggregations of ≥100 Townsend’s big-eared bats were observed at 41 different caves or mines across 9 states. We used zero-inflated negative binomial regression to explore biogeographic patterns of winter roost counts. *Myotis* counts were greater in caves than mines, in more recent years, and in more easterly longitudes, northerly latitudes, higher elevations, and in areas with higher surface temperatures and lower precipitation. Townsend’s big-eared bat counts were greater in caves, during more recent years, and in more westerly longitudes. Karst topography was associated with higher Townsend’s big-eared bat counts but did not appear to influence *Myotis* counts. We found stable or slightly-increasing trends over time in counts for both *Myotis* and Townsend’s big-eared bats from 82 hibernacula surveyed ≥5 winters since 1990. Highly-dispersed winter roosting of *Myotis* in the western USA complicates efforts to monitor population trends and impacts of disease. However, our results reveal opportunities to monitor winter population status of Townsend’s big-eared bats across this region.

## Introduction

Effective conservation relies on knowledge of the whereabouts and ecological needs of animals during critical phases of their annual cycle. Most species of bats in temperate areas spend half or more of each year in hibernation, which allows them to avoid seasonal prey shortages, limit mortality due to concealment and inactivity, and reduce somatic degradation [1]. Hence, availability of winter roosts has long been understood to contribute to patterns of bat community diversity [2]. Hibernation has been an evolutionarily successful strategy for temperate-zone bats, allowing them to persist in cold regions with net-positive population growth rates despite low fecundity and other “slow” life history traits relative to other small mammals [1, 3]. The central importance of hibernation to temperate-zone bat biogeography underscores the need to understand bat overwintering ecology [4]. However, our ability to do so is hampered in many regions by a fundamental lack of information about where bat hibernacula occur [4].

A prominent example of this is in the western United States, where the winter roosts of bats are poorly known [5–7]. Bats are ubiquitous in natural areas throughout the western USA, which commonly support multiple species of bats, and their activity patterns and roost habitats during summer have been the topic of extensive research [8, 9]. This discrepancy between the omnipresence of many species of bats during summer and dearth of information on their winter whereabouts throughout the western USA has long puzzled biologists (e.g., [7]). The prominent exception is for Townsend’s big-eared bats (*Corynorhinus townsendii*; hereafter COTO) which occur in conspicuous aggregations, mostly in caves and mines, during summer and winter [10–13]. Several recent studies have reported on multi-year monitoring efforts of COTO hibernacula in caves in the Pacific Northwest [14–16]. Winter roosts of big brown bats (*Eptesicus fuscus*) have been documented in rock crevices in Alberta, Canada [17, 18]. However, information on other species of bats during winter in western North America is largely limited to reviews of winter records [19–21] and reports of bat activity [18, 22, 23].

This lack of information about other species, especially from the genus Myotis, has been of increased concern in recent years given the devastating population impacts of white-nose syndrome (hereafter WNS) which affects bats during hibernation. The outbreak of WNS represents a major threat to multiple bat species because it greatly increases mortality rates, particularly among adults, over the generally low rates thought to be typical during hibernation [1]. As of August 2018, WNS has been confirmed in 11 species of bats in North America and, regionally, has devastated populations of several *Myotis* species [24–26]. The disease was first observed in a cave in New York in 2006 [27] and in subsequent years spread from its presumed point of introduction into adjoining states and Canadian provinces [24, 28, 29]. The disease is caused by the fungus *Pseudogymnoascus destructans* that thrives in cold, wet conditions typical of caves [30–32]. Accordingly, efforts to document impacts of the disease and detect its causative agent have primarily focused on surveillance of caves and mines [26, 33].

The impacts to bats in eastern North America were relatively easy to discern because locations of hibernacula were fairly well known and hibernating bats aggregated conspicuously within them [4, 34]. Population-level impacts have been quantified by comparing impacted population sizes against reconstructions of pre-disease population sizes [24, 26, 33, 35]. In fact, the outbreak of WNS appears to have precipitated the first regional estimates of population size for some species of bats [13, 24, 26]. Although a comprehensive database of hibernacula counts was lacking, regional estimates in these studies were possible due to similarities in census methods among states and hibernation behavior of bats within their winter roosts. In particular, many of the affected species tended to roost in large aggregations on exposed surfaces in caves and mines, which made them relatively easy to count.

During the decade-long westward encroachment of WNS there were efforts to describe and convey the dispersed wintering habits of western bat species and resulting challenges of conducting disease surveillance under these circumstances [36, 37]. However, as long as WNS stayed in eastern North America, it was difficult to inspire the institutional urgency needed to compile the survey records necessary to generate baseline assessments of population status for hibernating bats across the western USA. This changed in 2016, when a little brown myotis (*Myotis lucifugus*) with WNS was discovered in western Washington approximately 1300 km west of the nearest previous detection of the fungus or disease [29]. This jump in the distribution of WNS was unprecedented since its introduction into North America as spread of the disease had previously only occurred between adjoining states or provinces. This jump-dispersal event outpaced epidemiological models that forecast expected arrival in the Pacific Northwest (e.g., [38]), triggering land management and conservation organizations across the west to accelerate surveillance and monitoring activities [39–41]. However, given the different set of species that occur in the West, the vast and rugged landscapes to be searched, and presumed differences in overwintering behaviors of bats in western states [21, 23, 42], emergence of WNS in the Pacific Northwest necessitates a reevaluation of disease transmission pathways as well as the methods and strategies used to date to detect the disease, its causative agent, and its population impacts on bats.

As a first step towards addressing these needs we compiled historical and contemporary records of winter roost surveys conducted across the western USA. Our primary question was: What do we know about the winter roost habits of bats in the western USA and how confident are we they have been reliably characterized? Hence the objectives of this paper were to summarize the current state of knowledge of winter hibernation sites in the western USA including: quantity of survey effort, spatial distribution of survey effort, types of structures used by bats, species detected, size of aggregations, temporal trends in winter counts, and distribution of winter counts along elevational and other important biogeographic gradients. We constructed a large dataset for this effort by canvassing state and federal agencies across 11 states for existing data on bat hibernacula and survey effort during winter. We anticipated records of survey effort would vary among states and that most records would report counts or occurrence of bats but not results of surveys in which bats were not detected. We further anticipated that most survey effort would have focused on caves and mines, aggregations of bats would be smaller than those observed in the eastern USA, and the most common species and largest aggregations would be of COTO. We used the resulting dataset to explore biogeographic trends in hibernacula counts along environmental gradients using regression modeling techniques that may be useful to guide future disease surveillance and other bat conservation activities. We also used a subset of the data representing sites where bats had been counted over multiple years to assess evidence of temporal trends in hibernacula counts.

## Materials and methods

### Data compilation

We requested data on winter survey records for bats from 11 states: Arizona, California, Colorado, Idaho, Montana, Nevada, New Mexico, Oregon, Utah, Washington, and Wyoming. Our primary requests were sent to state wildlife management agencies, natural heritage programs, and state bat working group contacts that were part of the larger Western Bat Working Group network (http://wbwg.org). We also targeted data requests to the USDA Forest Service, Bureau of Land Management, National Park Service, and individual biologists following recommendations from primary requestees or when the number of records received following our primary request was lower than expected. We focused on detections of species in the genus *Myotis* due to their demonstrated susceptibility to WNS and also on detections of COTO, primarily because this species has been the focus of winter surveys across the western USA [43, 44]. We provided a template spreadsheet that prompted respondents to report structure name, structure type (e.g., cave, mine, building), survey date, survey location, bat presence/absence, count of COTO, count of *Myotis*, count of species other than COTO or *Myotis*, total count of bats observed during a survey, and any comments about the survey. Counts or presence of other species were sometimes included, often as comments.

We considered any survey conducted during the months of November through March to be a winter survey. We assigned each survey to the year in which the winter period ended. Structure type and geographic coordinates were sometimes not reported, but we retained these observations and compiled remaining information. We designated each observation as either a count or presence-only survey. A survey was designated as presence-only when it was originally reported as such, based on interpretations of the comments accompanying the survey, or, conservatively, when it was not possible to tell whether a value of 1 for a particular species group represented a true count or was simply a designation of presence. Records that reported zero bats observed were considered counts. When an observation reported presence of multiple species of bats but only a total count for the number of bats, we split the observation into 2 records: a count record that reflected total number of bats observed and a presence record indicating which species were present. We pooled reports of any species of *Myotis*, confident that most observers would be able to correctly identify bats to the genus-level but not consistently to species, many of which are cryptic even in the hand [45–47].

Spatial resolution of the locations provided for individual structures varied among and sometimes within states. Precise coordinates for individual structures were the most common resolution provided. Some jurisdictions were concerned about providing precise coordinates; for example, some national parks only provided locations for an area within the park where structures occurred. In these cases, a single set of coordinates, representing the centroid of the surveyed area, was used for multiple structures in our compiled database. The Federal Cave Resources Protection Act of 1988 and other federal and state regulations prevent public disclosure of exact cave locations (see [48] for a review).

### Data subsets

We explored biogeographic (spatial) patterns of hibernacula counts along broad environmental gradients with a subset of data consisting of a single representative survey per structure. We only considered surveys conducted in caves and mines from years 1990–2017, as older data and data from other structure types were sparse, and had inconsistent locational and other metadata. We generally selected the most recent survey where bats were detected to represent the count for each structure in the spatial analysis. However, if the most recent survey in which bats were detected was earlier than 2006, we used the most recent non-detection survey to represent the structure. We allowed the individual survey that represented the structure to differ according to whether the analysis was for COTO or *Myotis*. For example, if no *Myotis* were observed during the most recent survey of a structure in which ≥1 COTO was counted, yet ≥1 *Myotis* were counted during another survey after 2006, the most recent survey was selected to represent the structure for COTO and the earlier survey represented the structure for *Myotis*. In 98% of cases, results from the same survey represented the structure for both species. We explored temporal trends in hibernacula counts over time with a different subset of data consisting of replicate counts spanning ≥5 winters conducted in any structure type (e.g., buildings in addition to caves and mines) since 1990. When ≥1 count per winter was conducted, we selected the largest count for inclusion in trend analysis.

### Statistical analyses

We explored spatial and temporal trends in the data subsets for *Myotis* and COTO with zero-inflated negative binomial regression [49]. In our dataset, most counts were recorded at or near zero but some large counts were also reported that extended the distributional tail far to the right. The negative binomial distribution (a link function) provided a more suitable latent model for the over-dispersed (right-skewed) distribution of counts than alternatives including the more familiar Poisson [49]. However, the number of zeros in analyzed subsets were much greater than could be explained with the negative binomial distribution (zero inflation), regardless of the distributional dispersion parameter assumed. Hence we used zero-inflated negative binomial regression, which models zeros as a mixture of binomially-distributed probabilities via a logit link function and negative-binomial counts, some of which could also be zero [49, 50]. We assumed excess zeros in the dataset to be both structural, resulting from our incomplete understanding of winter hibernacula habitat use (i.e., some caves and mines were not used by bats for reasons we do not perceive), and from random variation (i.e., zeros recorded when a structure is suitable but not used), as well as due to imperfect detection. There was not enough information in the dataset to enable formal estimation of detectability (e.g., too few structures with repeat visits within winters).

Our biogeographic (spatial trend) models included covariates for survey year, structure elevation (elevation), mean annual temperature (temperature), mean annual precipitation (precipitation), karst topography (karst), and whether the structure was a cave or mine (mine). Karst was constructed as a binary indicator variable and year, elevation, temperature, and precipitation were centered at zero and standardized to improve computation and interpretation [51, 52]. Count coefficients, when exponentiated, are interpreted as the estimated multiplicative (percent) change in mean count. In our models, with survey data beginning in 1990, the average winter survey occurred in 2008 with a standard deviation of 7 years, and model parameters should be interpreted accordingly (e.g., model intercept is the mean count in 2008 at average elevation, precipitation, temperature, and on non-karst). We constructed and fit zero-inflated negative binomial regression models using the *pscl* package [53] in the R statistical environment [54].

We extended our negative binomial regression models with a random effect (separate intercepts and slopes) for individual structures to account for repeated counts at individual structures over time (and presumably not independent). We only included year as a covariate, focusing on the conservation concern of potential decline in counts over time across the collection of structures within our dataset that had ≥5 replicate counts. We fit these zero-inflated negative binomial mixed-effects models using the R package *glmmADMB* [55].

#### Biogeographic model covariates

We used a GIS to extract 30-year mean temperature and precipitation, elevation, and karst topography for each cave and mine location used in spatial trend models. Gridded climate data were the PRISM Climate Group’s 30-year normals for the period 1981–2010 [56]. Elevation data were from the U.S. Geological Survey National Elevation Dataset (now the National Map) at 30-m resolution (1 arc-second; https://nationalmap.gov/elevation.html). Karst and pseudo-karst (including volcanics) topography data were derived from Weary and Doctor [57]. For Colorado records, we instead used a detailed karst map that was used by the Colorado Bat Conservation Plan [58].

### Conservation risk assessment

To assess protection status of hibernacula, we overlaid hibernacula locations with the Protected Areas Database of the United States (PAD-US; [59]). The inventory provides 4 ranks according to conservation and access for public recreation as follows: 1) managed for biodiversity–disturbance events proceed or are mimicked; 2) managed for biodiversity–disturbance events suppressed; 3) managed for multiple uses–subject to extractive (e.g., mining or logging) or off-highway-vehicle use; 4) no known mandate for protection. Some locations were not ranked so we assigned these to the 4th rank. We also overlaid hibernacula locations with the gravity_caves_+ winter maximum likelihood predictive model of WNS spread produced by Maher et al. [38] to assess the pattern of *Myotis* hibernacula size in relation to WNS arrival risk.

## Results

We compiled results of 4,549 winter surveys for bats from 2,888 unique structures across 11 states that were reported between the winters of 1916 and 2017 (Table 1). Across all states we categorized 78% of surveys as counts and 22% as presence surveys. Among the surveyed structures for which type was reported, 97% occurred at either caves (39%) or mines (58%); however the relative composition varied among states (Table 1). Mines represented >90% of structures surveyed in Colorado, Nevada and Utah whereas caves represented >83% of structures surveyed in Arizona, Idaho, and New Mexico. Bridges (*n* = 51, 41 of which were in Oregon) were the only other structure type that represented >1% of the total structures surveyed.

**Table 1:** Winter survey effort for bats from 11 states. Number of caves and mines represent minimum estimates of the number of unique structures reported in databases. Number of surveys is greater than sum of unique structures surveyed because some sites were surveyed multiple times.

State	Year of Earliest Record	Year of Latest Record	Unique Number of Years	90% Survey Effort (yrs)	No. of Surveys	No. of Counts	No. of Presence Surveys	No. of Caves	No. of Mines	Other Structure Types	Unknown Structure Type	No. of Unique Structures
AZ	2006	2014	6	2008–2014	101	97	4	83	16	0	0	99
CA	1938	2017	43	1994–2017	1,288	988	300	350	188	10	23	571
CO	1958	2016	28	2000–2016	627	627	0	24	538	0	0	562
ID	1932	2016	29	1996–2016	297	268	29	174	20	1	0	195
MT	1938	2015	27	1970–2015	104	97	7	41	26	1	0	68
NM	1967	2016	34	2002–2016	352	352	0	189	23	0	0	212
NV	1966	2017	11	2010–2017	412	181	231	21	292	3	0	316
OR	1916	2016	53	1986–2016	468	406	62	94	29	47	54	224
UT	1992	2017	22	2000–2017	390	95	295	7	340	0	0	347
WA	1938	2016	48	1978–2016	251	193	58	63	52	16	14	145
WY	1994	2016	18	1995–2016	259	258	1	38	103	8	0	149
**TOTALS**	1916	2017	73	1994–2017	4,549	3,561	988	1,084	1,627	86	91	2,888

**Fig 1:**
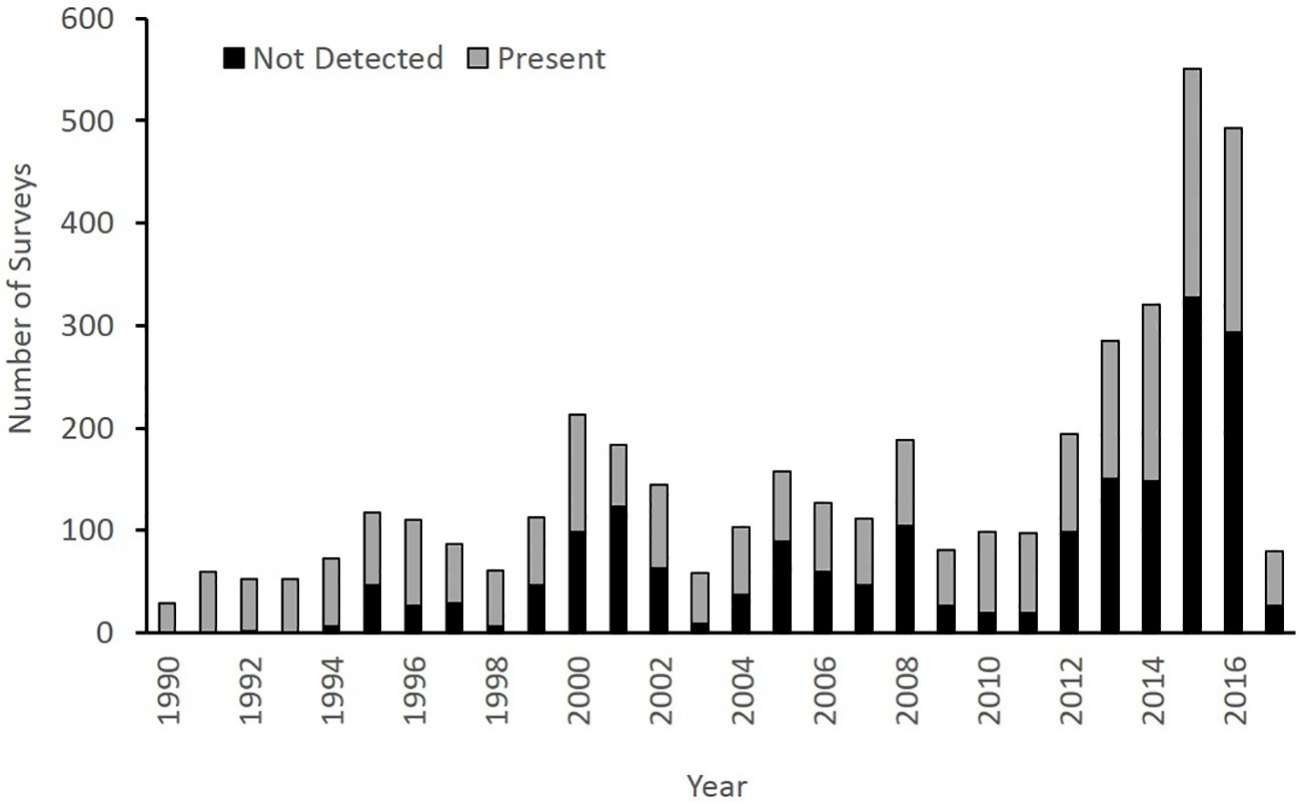
Number of winter surveys per year and proportion that reported bat presence since 1990 across 11 states. Note that most states only provided data compilations through 2016 so compiled data 2017 was incomplete.

Over 95% of recorded surveys occurred since 1990, reflecting an apparent increase in survey effort and improved record retention. Almost half (43%) of all surveys were compiled after 2011 (Fig 1). Prior to 1990, surveys in which bats were not detected represented only 2 of 217 records. Since 1990 the proportion of records that also recorded non-detection of bats rose steadily with non-detection surveys representing 16% of surveys from 1990–1996, 44% of surveys from 1997–2003 and 2004–2010, and 53% of surveys from 2011–2017.

Overall, *Myotis* were reported from 19% and COTO was found in 38% of structures searched across 11 states. The proportion of surveyed structures where *Myotis* were detected varied from 4% in Utah to 44% in Montana. The proportion of surveyed structures where COTO was reported ranged from 22% in Utah to 68% in Washington. In 61% of the structures in which *Myotis* were found, COTO was also found ranging from 39% of 139 structures in Colorado to 94% of the 62 *Myotis* hibernacula in Nevada. Across all states, *Myotis* were found in 19% of 1084 caves and 18% of 1627 mines (Table 2). COTO was detected in 44% of caves and 34% of mines across all states.

**Table 2:** Proportion of caves and mines occupied by *Myotis* spp. and *Corynorhinus townsendii* (COTO) and largest winter count recorded, by structure type, across 11 states.

	Caves					Mines				
State	Number of Sites	% with *Myotis*	% with COTO	Largest *Myotis* Count	Largest COTO Count	Number	% with *Myotis*	% with COTO	Largest *Myotis* Count	Largest COTO Count
AZ	83	9.6	31.3	500	200	16	18.8	25.0	1	27
CA	350	14.3	40.0	22	699	188	13.3	66.5	8	80
CO	24	25.0	66.7	23	605	538	24.7	30.7	13	190
ID	174	17.8	40.2	804	1,932	20	40.0	80.0	4	83
MT	41	43.9	39.0	1,738	20	26	46.2	73.1	14	36
NM	189	11.6	24.3	4,962	1,468	23	13.0	13.0	5	24
NV	21	38.1	38.1	4	24	292	18.5	24.3	34	455
OR	94	38.3	75.5	79	356	29	6.9	86.2	2	28
UT	7	42.9	85.7	3	385	340	2.9	20.6	6	79
WA	63	20.6	92.1	12	300	52	11.5	50.0	6	50
WY	38	36.8	44.7	67	47	103	34.0	33.0	33	54
**TOTALS**	1,084	19.3	43.6	4,962	1,932	1,627	17.8	34.3	34	455

Bats other than COTO and *Myotis* were reported from 267 surveys (6%), 248 of which were counts. The most frequently reported “other” species was big brown bat. Single individuals represented 61% of the nonzero counts of other species, 88% were counts of ≤3 individuals and 93% reported groups of ≤5 individuals.

### *Myotis* group sizes

**Fig 2:**
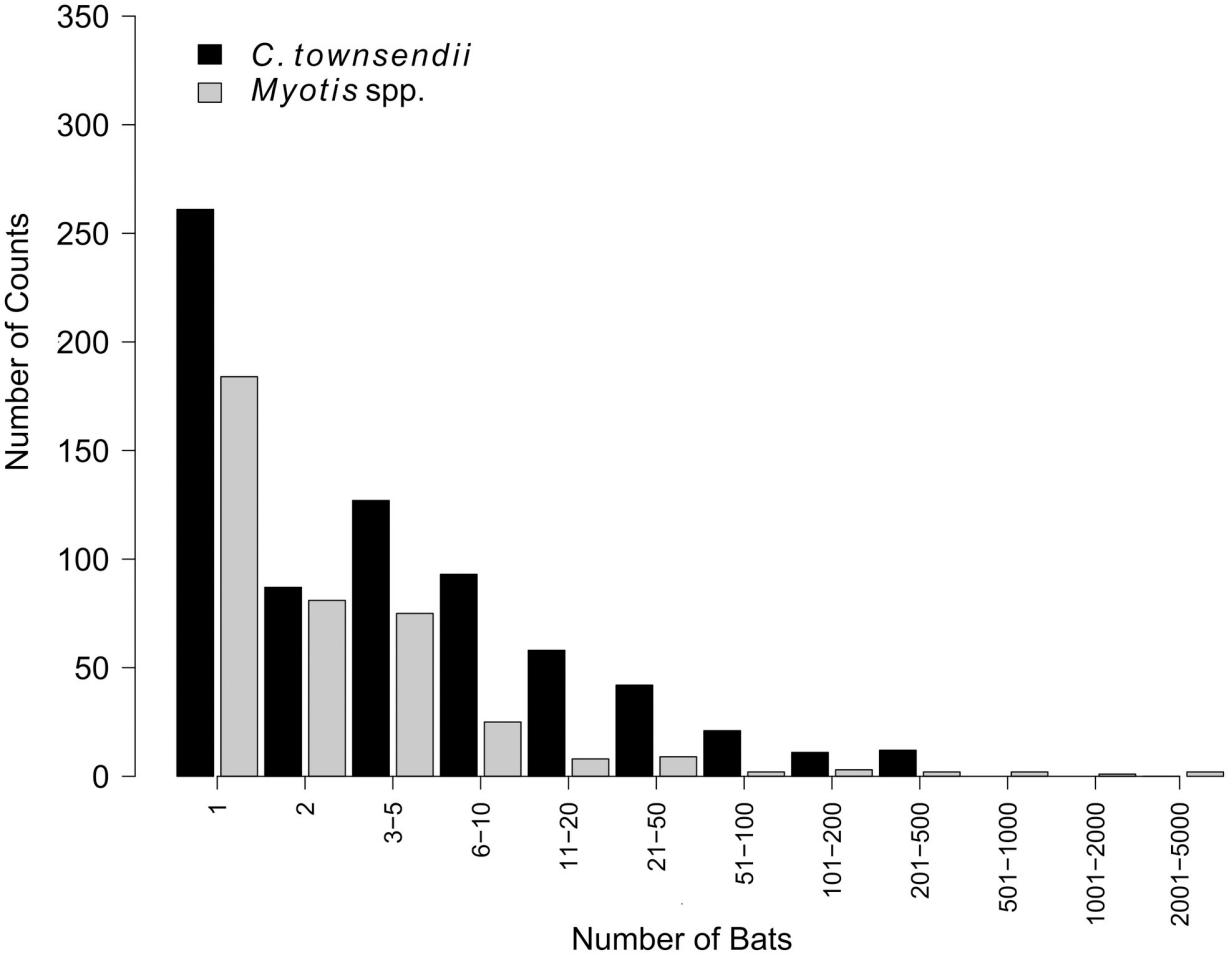
Frequency of non-zero counts of *Corynorhinus townsendii* (COTO) and *Myotis* spp. reported from hibernacula across 11 western states since 1990. A single, usually the most recent, count was selected to represent each structure.

**Fig 3:**
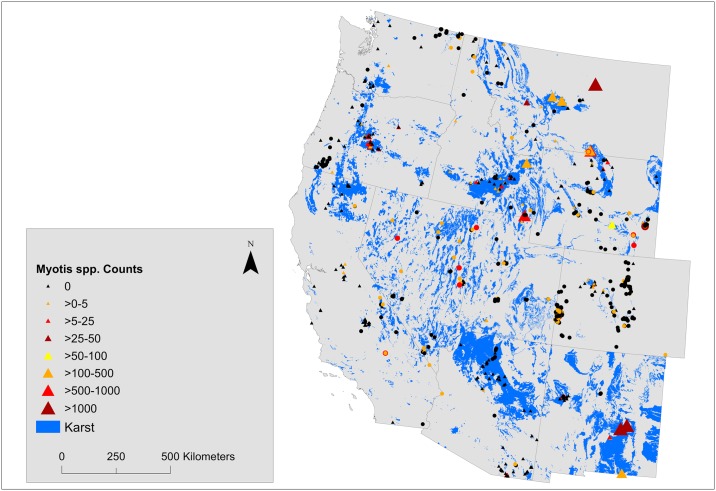
Hibernacula counts of *Myotis* spp. in 11 states overlain on maps of karst topography. Caves are depicted as triangles and mines as circles.

Median nonzero *Myotis* group size was 2 individuals across all states, ranging from 1 individual in 3 states to 5 individuals in New Mexico (S1 Fig). Single bats accounted for 45% of nonzero counts of *Myotis* in caves, 86% of observations were of aggregations of ≤5 individuals, and 95% were of ≤10 individuals. Large aggregations of *Myotis* (>500 individuals) were reported from caves in 4 widely separated states (Arizona, Idaho, Montana, New Mexico; Figs 2 and 3). In all states except Nevada, the largest winter aggregations of *Myotis* occurred in caves rather than mines. Idaho reported aggregations of 804, 146, 52, 25, and 24 *Myotis* from 5 separate caves but the remaining 35 structures each contained ≤10 individuals. Montana reported maximum counts of ≥200 wintering *Myotis* from 5 separate caves. New Mexico reported *Myotis* hibernacula in 3 caves containing 337–4,962 individuals, while the remaining 22 structures surveyed in New Mexico had maximum *Myotis* aggregations of ≤18 individuals, 20 of which had ≤10 individuals. In the remaining 7 states, the largest aggregation of *Myotis* was 79 individuals in Oregon (Table 2).

Single bats represented 51% of nonzero counts of *Myotis* in mines. Of these 94% of counts were of aggregations of ≤5 individuals, and 97% were of ≤10 individuals. The largest aggregations of *Myotis* in mines were of 34 individuals in Nevada and 33 individuals in Wyoming. Nevada reported 6 of the 11 mines in which ≥10 *Myotis* were found wintering.

### *Corynorhinus townsendii* group sizes

**Fig 4:**
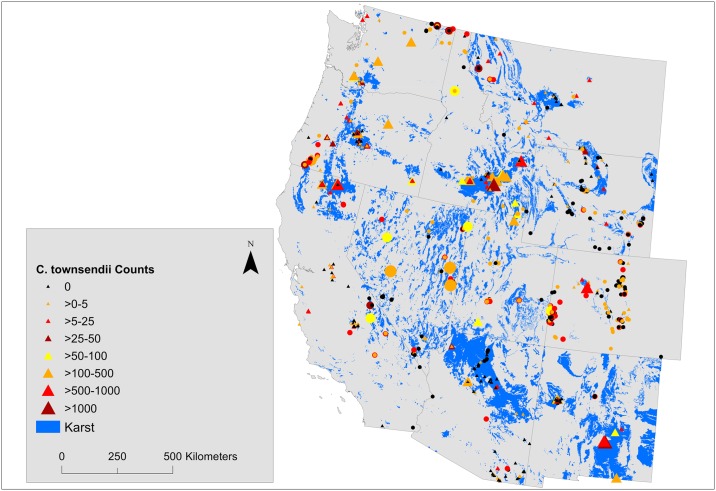
Observed hibernacula counts of *Corynorhinus townsendii* in 11 states overlain on maps of karst topography. Caves are depicted as triangles and mines as circles.

Median nonzero COTO group size was 3 individuals across all states ranging from 1 individual in Colorado to 6 individuals in Idaho (S1 Fig). COTO aggregations in caves were generally greater than in mines. Counts of >500 COTO were reported from 6 caves in 4 different states, the largest of which was 1,932 individuals from a cave in Idaho (Table 2, S1 Fig). Aggregations of ≥100 COTO were observed from 41 different caves across 9 states (Figs 2 and 4). Nevertheless most counts were small. Single bats represented 28% of nonzero counts of COTO in caves, 56% of observations were of aggregations of ≤5 individuals, and 72% were of ≤10 individuals. States with the smallest maximum group sizes of COTO were Montana (36 individuals) and Wyoming (54 individuals), both of which were in mines. COTO were found in aggregations up to 455 individuals during surveys of mines. Aggregations of ≥100 COTO were observed in 6 mines, 5 of which were in Nevada. Single bats accounted for 38% of nonzero COTO counts in mines, 71% of counts were of aggregations of ≤5 individuals, and 83% were of ≤10 individuals.

### Biogeographic model results

Our regression models estimated that mean counts of *Myotis* were greater in caves than mines, in more recent years, and in more easterly longitudes, northerly latitudes, higher elevations, and in areas with higher mean annual surface temperatures and lower precipitation (Table 3). Mean counts of COTO were also estimated to be greater in more recent years, in cave locations within karst habitat, and in more westerly longitudes (Table 3).

**Table 3:** Regression model coefficient estimates for hibernacula counts of *Myotis* spp. and *Corynorhinus townsendii* compiled across 11 western states from 1990–2017. A single, typically the most recent, count per structure was used in the models.

***Myotis* spp**.			
**Parameters**	**Coefficient**	**SE**	**P value**
Intercept	1.89	0.32	<0.01
Mines	-2.90	0.32	<0.01
Year	0.35	0.09	<0.01
Elevation	0.73	0.35	0.03
Karst	0.37	0.31	0.21
Latitude	1.03	0.34	<0.01
Longitude	1.05	0.12	<0.01
Precipitation	-0.33	0.12	0.01
Temperature	1.11	0.36	<0.01
***C*. *townsendii***			
**Parameters**	**Coefficient**	**SE**	**P value**
Intercept	2.55	0.20	<0.01
Mines	-1.53	0.20	<0.01
Year	0.32	0.06	<0.01
Elevation	-0.01	0.21	0.92
Karst	0.39	0.19	0.04
Latitude	0.01	0.21	0.96
Longitude	-0.35	0.10	<0.01
Precipitation	0.00	0.06	0.98
Temperature	-0.03	0.22	0.87

**Fig 5:**
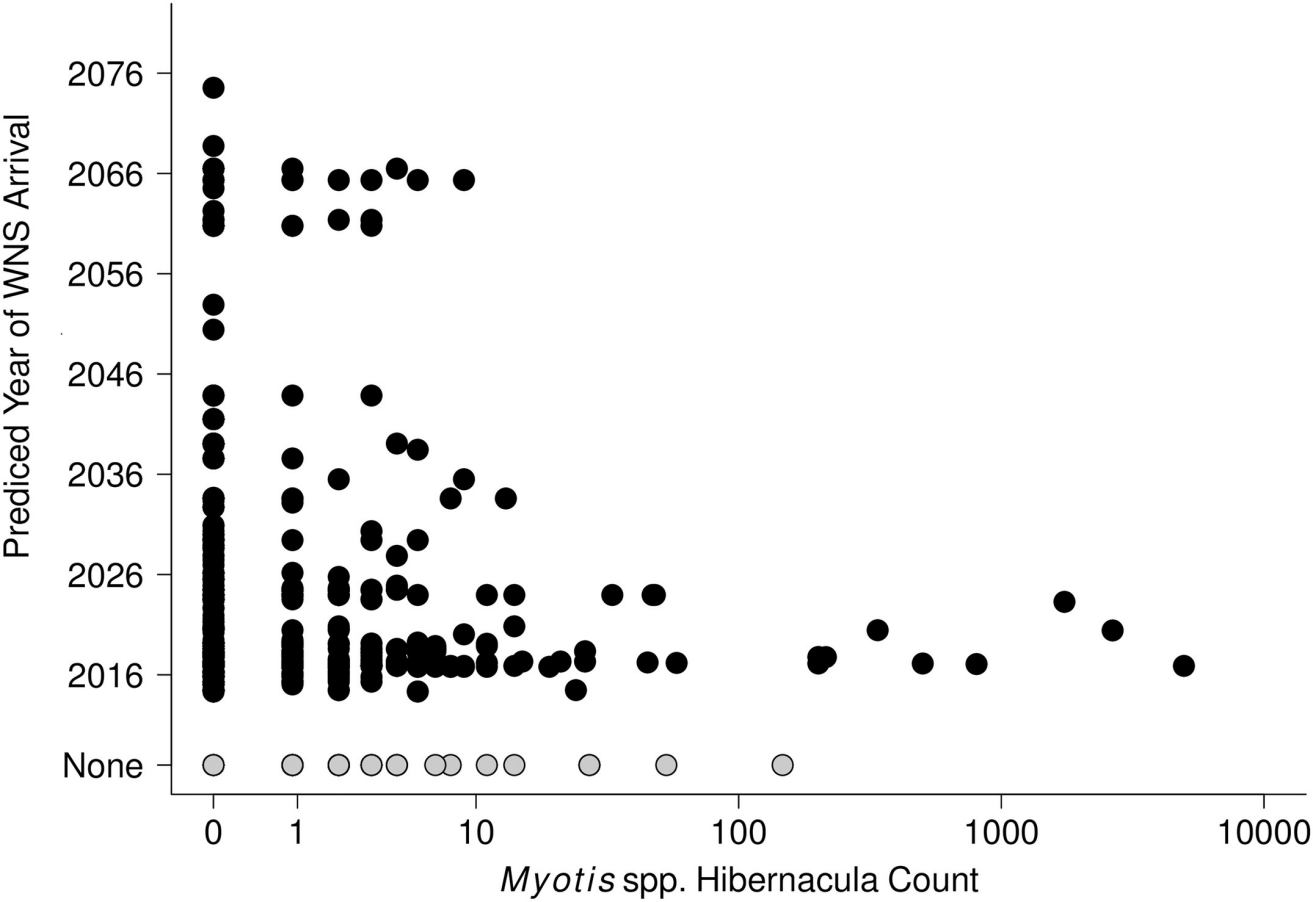
Size of *Myotis* spp. hibernacula versus predicted year of WNS infection based on Maher et al. [36].

The 25 largest *Myotis* hibernacula (≥20 bats) all occurred on lands in the lower 2 tiers of protected area status (status 3 and 4; [59]) including the largest, which was in the lowest tier but was on State Trust Lands (S2 Fig). By contrast, the largest COTO hibernacula were distributed across areas with conservation status 2–4 (S2 Fig). Tier 1 areas with the most stringent conservation protections hosted relatively small hibernacula of both COTO (≤20 individuals) and *Myotis* (≤10 individuals). We found 9 of 10 largest *Myotis* hibernacula were in areas predicted by Maher et al. [38] to experience earlier arrival of WNS (Fig 5, S3 Fig). Twenty-three of the 31 largest *Myotis* hibernacula occurred in areas predicted to have WNS exposure by 2025; the remaining 8 were in areas with no predicted WNS establishment.

### Temporal trends in counts

We identified 82 individual structures in which ≥5 counts had been recorded since 1990 including 74 caves, 5 mines, 2 buildings and 1 bunker. All states except Arizona and Montana reported at least 1 structure with ≥5 repeat counts (S4 Fig). Repeat counts were conducted at 46 structures in California (44 of them in Lava Beds National Monument) and 16 structures in Oregon. We found evidence for nearly flat (all <1% per year positive rate of change) temporal trends in counts of COTO and *Myotis* from structures surveyed during ≥5 winters (S5 and S6 Figs). The temporal trend for COTO from the negative-binomial model with random effects was 0.38% per year increase (*p* <0.001). The trend for *Myotis*, after removing 23 structures where *Myotis* had never been detected, was similar at ~0.30% per year (*p* = 0.0078).

## Discussion

Efforts to conserve bats in the western United States have long been impeded by a lack of knowledge of the winter whereabouts of most species. Our results suggest that this has not been for lack of survey effort, at least in caves and mines. We found there has been considerable effort, particularly in the recent past, to survey winter hibernacula of bats in western states. Our database revealed that during 1995–2016 an average of at least 177 surveys per year were conducted across the region and during 2012–2016 the annual average was 369 surveys per year. Only 3 of the 20 hibernacula (15%) that contained ≥30 *Myotis*, but 30 of the 92 COTO hibernacula (33%) had first records in our database after 2011. This suggests that that recent increased survey effort has generally been effective for locating large COTO hibernacula but less effective for *Myotis* hibernacula. Survey effort has been relatively well-dispersed geographically and, at least for caves, has focused on areas of karst and volcanic habitat. Encouragingly, most states have been tracking negative surveys, at least in recent years, as opposed to only those surveys where bats had been detected (Fig 1). The inclusion of non-detection records greatly facilitated our biogeographic trend modeling. Structures with more bats tended to be surveyed over a greater number of years. This trend is consistent with patterns observed in northeastern USA and Europe [35] and represents a frugal approach to monitoring for conservation.

We found no evidence of decline in the numbers of bats being counted in routinely surveyed winter roosts, a reassuring finding given that survey effort and tracking of survey results has improved in recent years while the threats facing bats in the western USA, and elsewhere, are increasing [4, 6, 60]. However, it is important to note that most surveys were from either caves or mines. This reflects a bias in that these were where most biologists expected to find hibernating bats during winter. Surveys of caves and mines in the eastern USA have been invaluable for documenting the population status for multiple species of bats including several endangered species [24–26, 33, 61]. However, inference from the data we assembled is that for most *Myotis* and likely other species (e.g., big brown bats) most individuals do not use caves and mines as winter roosts in the western USA.

### *Myotis* hibernacula

Despite extensive effort to search for bats during winter and our effort to federate these data into a single regional database, the winter whereabouts of *Myotis* across much of the western United States remains enigmatic. As expected, *Myotis* were not detected in most structures surveyed and were found in low numbers when they were detected. There were only 11 records of structures that contained ≥100 *Myotis* and 6 structures that contained ≥500 individuals. Hence our work reinforces results from earlier studies that across the western USA most *Myotis* individuals do not hibernate in caves and mines [7, 21]. This pattern contrasts strongly with records of *Myotis* hibernacula in caves and mines of the eastern United States which, prior to the arrival of WNS, often contained thousands of bats [24–26]. We suggest that differences in wintering behavior between eastern and western *Myotis*, rather than lack of survey effort, explains the paucity of large winter aggregations of *Myotis* in caves and mines of the western USA. However, our analyses grouped multiple species from the genus Myotis into a single analysis group, which undoubtedly obscured important differences in ecology and behavior and impedes further speculation on differences in hibernation behavior among species.

Although median colony sizes of *Myotis* were small, our models showed a positive association with year indicating that reported colony sizes have been larger in recent years. However, it is unclear whether colony sizes are truly increasing or whether higher counts in our study resulted from a combination of improved survey effort and better record keeping in recent years. Nevertheless, this result was supported by our analysis of 59 individual structures surveyed during ≥5 winters that also showed a stable-to-slightly increasing trend in colony size of similar magnitude. Hence, best available evidence suggests that *Myotis* populations that hibernate in caves and mines in western states were stable prior to the invasion of WNS. Although our analyses indicate that few of the largest *Myotis* hibernacula are currently within protected areas, multiple options are available to protect bats that roost in caves and mines (e.g. gating, seasonal closures).

Our biogeographic models indicated that *Myotis* counts trended statistically along several of the biogeographic variables examined. An association with eastern longitudes was evident, as by far the largest hibernacula occurred along the eastern extent of our study range. In New Mexico, hibernacula were composed of *Myotis velifer* that roosted in large numbers in exposed locations within caves. In Montana, the large *Myotis* roosts were thought to be composed largely of *M*. *lucifugus*, which also roosted in exposed locations within caves [62]. The winter roost behavior of *M*. *velifer* in New Mexico and *M*. *lucifugus* in the northeast portion of our study area contrasts strongly with data from *Myotis* counts elsewhere in the western USA which were typically small numbers of individuals in crevices within roost structures. This east-west trend in colony size may reflect the increased genetic differentiation that occurs within western populations of *M*. *lucifugus* relative to eastern populations [63, 64], or it may be a behavioral response to past population stressors that reduced populations or selected for more dispersed winter roosting behavior [35]. Alternatively it may be due to differences in winter roost habitat availability.

After accounting for associations between higher counts of *Myotis* with more northerly latitudes and higher elevations, our spatial model also indicated an apparent association with higher surface temperatures and lower precipitation. We used coarse-scale gridded surface temperatures derived from interpolation among weather stations [56], which is decoupled from the actual temperatures experienced by bats within caves and mines during hibernation. As a rule of thumb, cave temperatures are thought to reflect mean annual surface temperatures; however, the microclimate of caves and mines are determined by a complex suite of factors including number, size, and orientation of openings, depth, and airflow patterns [65, 66]. Future efforts that are able to establish actual internal winter temperatures across a large geographically-representative sample of hibernacula structures (e.g., from caves, mines, and building) could provide stronger insights into the relationships between hibernacula size, temperature, and other hibernacula attributes.

### *Corynorhinus townsendii* hibernacula

We found COTO hibernacula to be well distributed throughout the species’ range in the western USA (Fig 4). COTO was found in more than twice as many structures as were *Myotis* and group sizes were consistently larger than for *Myotis*. Maximum group sizes we compiled are in accordance with historic estimates for COTO [43]. Smaller maximum group sizes for COTO in Montana and Wyoming are to be expected as this is where the western subspecies reaches the northeastern extent of its distributional range [67]. Counts of COTO were greater in caves than in mines which supports findings from northern Utah that caves represent a more suitable resource for this species [11]. Lava tube caves appear to be particularly important for the species in some regions [12, 13, 16].

Similar to *Myotis*, we found evidence that colony counts had increased in recent years, possibly as a result of increased effort and improved record keeping, and we found stable-to-slightly increasing counts over time at roosts that had been monitored over multiple years. Hence, indications are that COTO population status across the region has been stable despite the relatively low proportion of hibernacula that occur on formally protected lands. Past concerns about the population status of COTO (e.g., [44]) were likely the primary motivator of hibernacula surveys in western states prior to the current era of WNS. Several studies focused on single structures and smaller geographic extents have also found COTO trends to be stable to increasing at individual sites [14–16]. Our results expand on these narrower inferences with reassuring findings of apparent population stability range-wide. However, it is important to underscore that our inferences are still constrained by a non-random, albeit large, sample with unknown representation of the true distribution and size of the western COTO hibernacula population. Based on our findings, and in contrast to *Myotis*, we consider it likely that additional medium to large COTO hibernacula remain to be discovered in the western USA.

### Characteristics of caves and mines used for bat hibernation

Although we did not find strong and consistent differences between the proportions of caves or mines that provided wintering habitat for at least single individuals, caves hosted larger winter aggregations of both *Myotis* and COTO than mines across the region. Nevertheless it is important to recognize that the impetus for surveys of caves was likely different than for mines and this may have resulted in dataset bias. Winter surveys of caves were likely motivated by suspected presence of bats, whereas winter mine surveys were often conducted to assess bat use to determine preferred timing and methods for closure; hence counts at caves may be biased high. For example, 96% of structures surveyed in Colorado and 98% in Utah were conducted at mines, many of which were likely selected based on their priority for closure as part of the Abandoned Mine Lands (AML) reclamation program, rather than an expectation that they contained large numbers of bats ([65,68], S1 Text). On the other hand, humans have created an abundance of cave surrogates over the past 150 years in the form of abandoned mines and some of them provide conditions bats seek for overwintering. As an example, the largest winter aggregations of COTO in Montana and Wyoming were in mines and we speculate use of abandoned mines may have allowed the species to extend the northeastern extent of its winter range.

Association of bat abundance and potential disease spread has consistently been related to presence of karst habitat, barren land, or rugged landscapes [35, 38, 69], which is not surprising since this is where most bats have been found during winter. We found an association between higher counts and karst habitat for COTO but not *Myotis*, but this may be driven by the large number of mines in our dataset, and the fact that only 24% of mines occurred in karst habitat. The karst layer we used for Colorado [58] demonstrated that the regional map of karst habitat we used [57] may be too liberal in its depiction of the type of karst habitat used by bats in other states. Use of higher resolution karst maps may help refine conclusions about karst association, at least for cave hibernacula.

### Prospects for winter hibernacula monitoring

For western *Myotis*, threatened with advancing fronts of WNS from both the Great Plains and western Washington, there is increased urgency to monitor populations and assess expected impacts of disease. Unfortunately few clear biogeographic trends emerged from our modeling efforts that can be used to guide future discoveries. Clearly, the few large *Myotis* hibernacula identified in our study should continue to be high priorities for monitoring and protection. Additional vigilance at hibernacula in areas where predicted arrival of WNS is earlier [38] is also justifiable. Nevertheless, with few exceptions, our results confirm that *Myotis* across the western USA do not occur in large aggregations; instead these bats likely hibernate individually or in small groups, in dispersed and inconspicuous locations. Although such roosting behavior may ameliorate the spread of WNS [70] it greatly complicates efforts to monitor population trends and impacts of disease. Alternative strategies could involve the use of radio-telemetry or other ways to locate inconspicuous crevice roosts (e.g. [17, 18, 23, 42, 71, 72]). However, because these methods are likely to reveal only small groups of bats, they may be ineffective for monitoring regional population trends or implementation of regional disease management strategies.

There appear to be more opportunities to continue to use hibernacula surveys of caves, mines, and buildings to monitor COTO trends over time. Future efforts should not only focus on the largest identified hibernacula but also prospect for additional large hibernacula. Furthermore, additional robustness to status and trend estimates could be introduced via statistical sample site selection. For example, a modified dual-frame sampling regime [73, 74] could be employed from which a list of known and accessible hibernacula comprises the list frame and an area frame is used for prospecting and adding new sites into the monitored population. The North American Bat Monitoring Program [75] provides a grid-based areal frame and randomized master sample that could be employed for this purpose.

### Data management

Posing similar questions to ours about lack of knowledge on hibernating bats, Twente [7] surveyed over 500 caves and mines and more than 100 buildings in northern Utah, however current databases do not contain these records. Similarly, few of the records from more than 700 structures surveyed in Oregon and Washington [21] or 31 caves surveyed in Idaho [76] during winters of the 1980s appear to have been archived, nor any of the 70 mines surveyed in Nevada during the mid-1990s by Kuenzi et al. [77]. Although, at least some records of mine surveys appear to have been retained, it is clear that many records from 820 structures surveyed in northern Utah during the mid-1990s, including 29 records of COTO wintering in caves [11], are not in current wildlife databases. We compiled few records from the 1970s (*n* = 45) and 1960s (*n* = 15) despite recent knowledge of some of these studies [43]. Given limited resources available for bat conservation, it is vital that survey results are archived, both to improve population status assessments over time as well as to prevent inefficiencies via unintended repeat surveys of unoccupied structures. Although efforts to compile results of roost monitoring efforts, including hibernacula, began as early as 1995 [43] an internet-accessible repository of monitoring results has not become a reality up to present. As an interim step toward this goal, we have archived a public version of our dataset (https://irma.nps.gov/DataStore/Reference/Profile/2247583), including covariates, for use by the scientific community. Our data archive removes structure names and provides spatial location precision resolved to the 100 km^2^ North American Bat Monitoring Program (NABat, [75]) sampling frame grid cell to prevent disclosure of exact locations of hibernacula. Ultimately, we advocate for creation of a database that allows users to upload new, and newly-unearthed historical records via a graphical interface that would allow contributors to archive their data in perpetuity. We acknowledge legal and proprietary concerns surrounding data sharing, particularly locations of sensitive underground sites and vulnerable bat populations, but argue the conservation community can no longer afford to lose occurrence records; a practice that impedes efforts to monitor and conserve bat populations over longer periods (e.g., decades to centuries). A comprehensive catalog of geospatial resources with existing and potential bat hibernacula would also be instrumental in developing an organized approach to monitoring population status of hibernating bats especially in the face of disease or other emerging threats [78].

## Supporting information

S1 Fig Box plots of non-zero counts of A) Myotis spp. and B) Corynorhinus townsendii in hibernacula across 11 states. A single, usually most recent, survey represents each structure. Boxes represent the 1^st^ and 3^rd^ quartiles, horizontal line represents median, whiskers represent 95% of the data and dots represent outliers for each state.
(TIF)

S2 Fig Size of hibernacula according to protected area status (Gergely and McKerrow 2016) across 11 western states. Symbols represent a single count from each structure with triangles representing *Myotis* spp. and circles representing *Corynorhinus townsendii*.
(TIF)

S3 Fig Observed hibernacula sizes of *Myotis* spp. overlain on a map of counties in 11 states. Counties are shaded according to predicted year of arrival of White-nose Syndrome (WNS) by Maher et al. (2012). Open red circle indicates King County, Washington where WNS was diagnosed in 2016.
(TIF)

S4 Fig Distribution of 82 hibernacula that were surveyed in ≥5 winters since 1990. (TIF)

S5 Fig Annual trends in counts of *Corynorhinus townsendii* from 82 hibernacula surveyed during ≥ 5 winters from 1990–2017 across 11 western states. (TIF)

S6 Fig Annual trends in counts of *Myotis* spp. from 82 potential hibernacula surveyed during ≥ 5 winters from 1990–2017 across 11 western USA states. Trends from a single hibernacula in New Mexico are plotted in sub-panel A while the remaining 81 structures are plotted in sub-panel B.
(TIF)

S1 Text Supplemental information. Descriptions of survey efforts by state.
(DOCX)
