# Supplementary figures and images for: A review of bat hibernacula across the western United States: Implications for white-nose syndrome surveillance and management
Source: PLoS One. 2018 Oct 31;13(10):e0205647. doi: 10.1371/journal.pone.0205647 (PMC6209190; doi:10.1371/journal.pone.0205647)

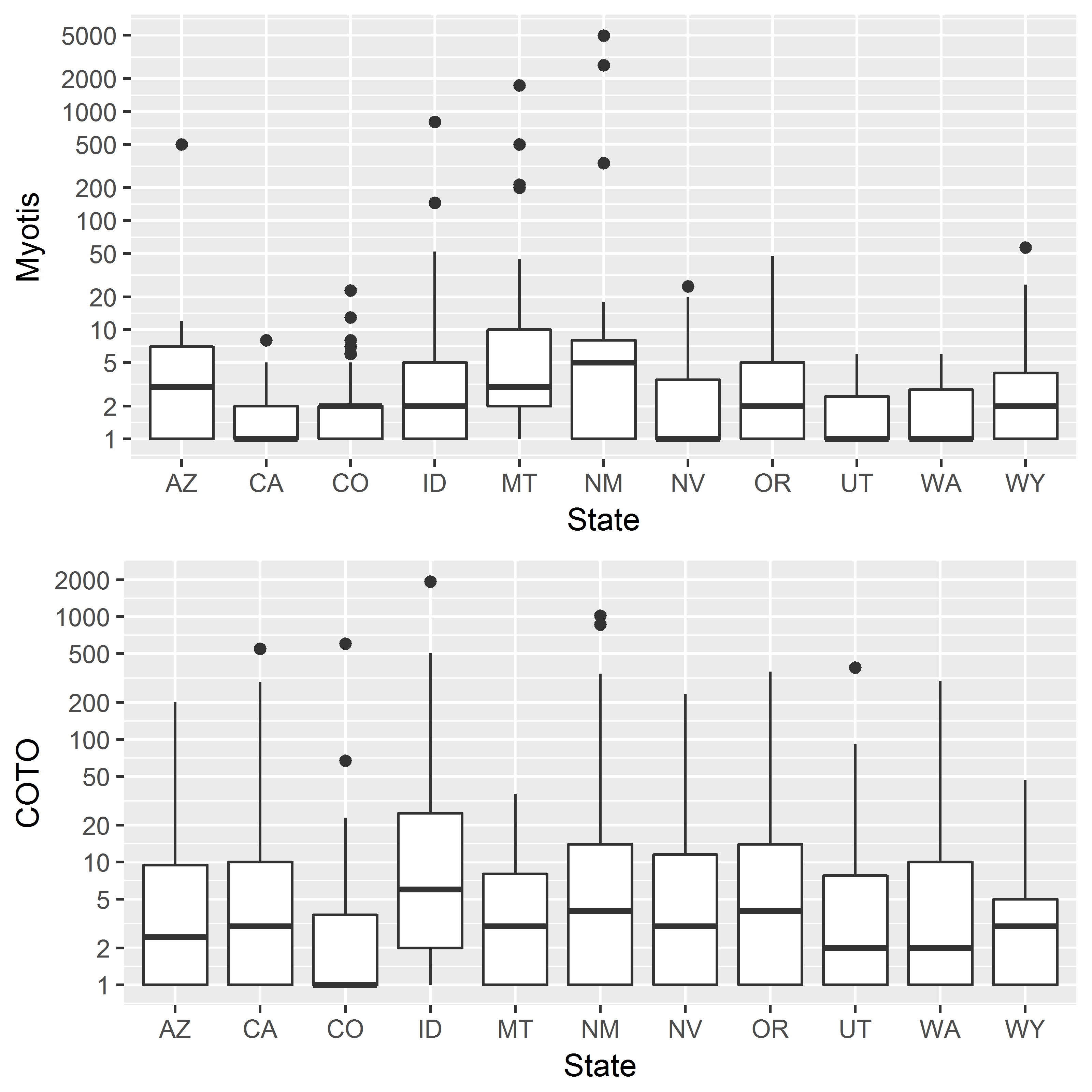

Supplement: S1 Fig — A single, usually most recent, survey represents each structure. Boxes represent the 1st and 3rd quartiles, horizontal line represents median, whiskers represent 95% of the data and dots represent outliers for each state. (TIF) [file pone.0205647.s001.tif]

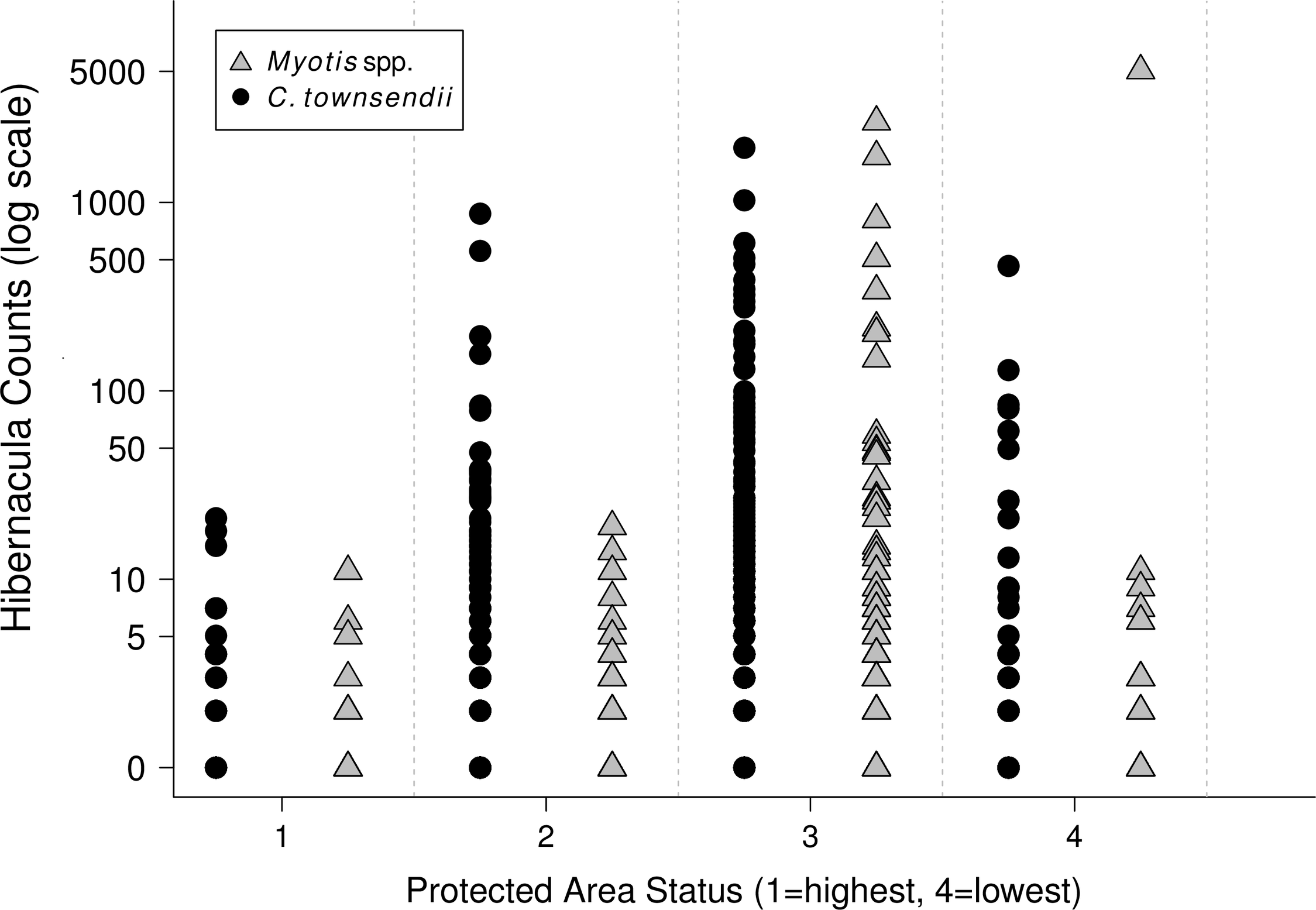

Supplement: S2 Fig — Symbols represent a single count from each structure with triangles representing Myotis spp. and circles representing Corynorhinus townsendii. (TIF) [file pone.0205647.s002.tif]

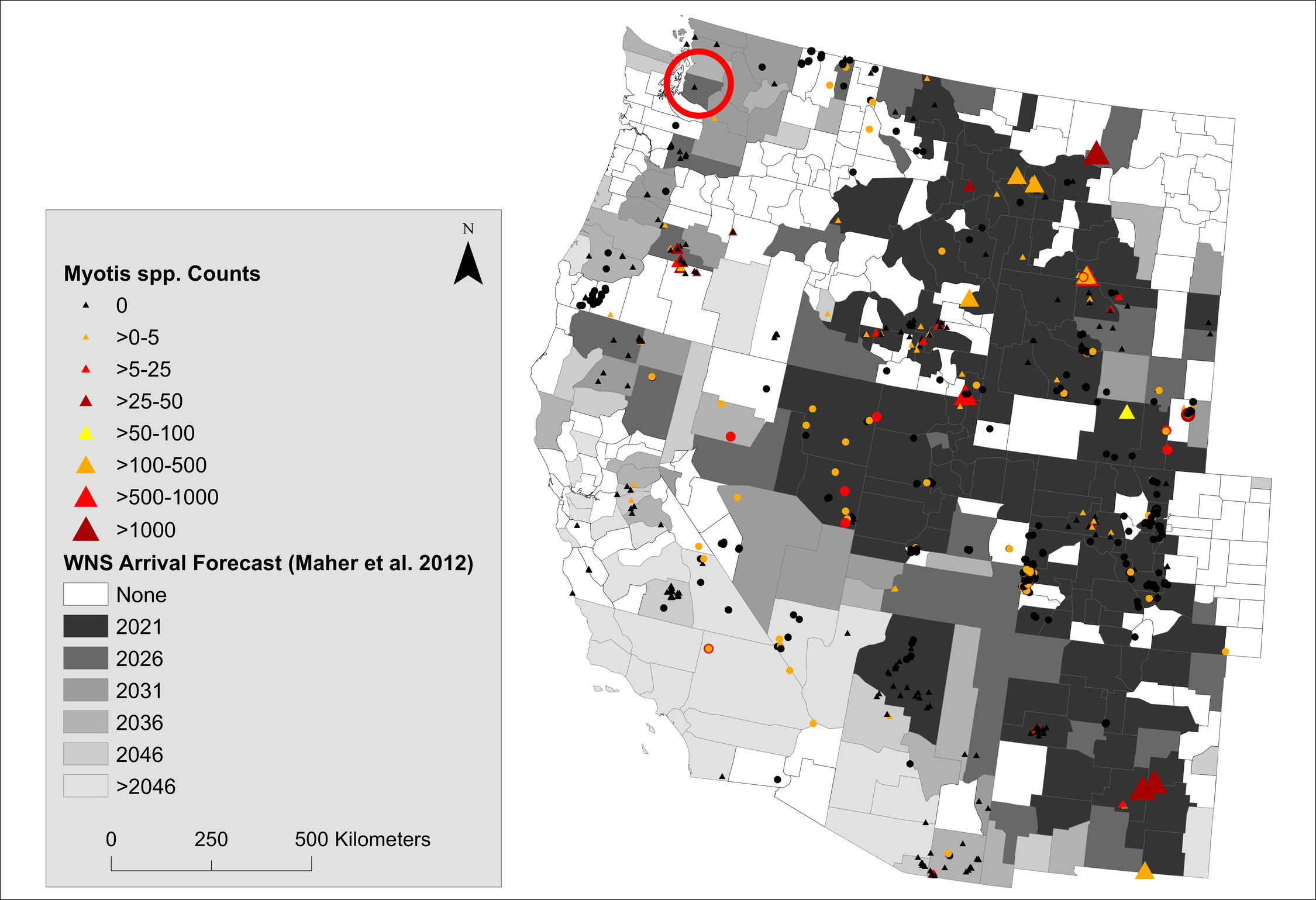

Supplement: S3 Fig — Counties are shaded according to predicted year of arrival of White-nose Syndrome (WNS) by Maher et al. (2012). Open red circle indicates King County, Washington where WNS was diagnosed in 2016. (TIF) [file pone.0205647.s003.tif]

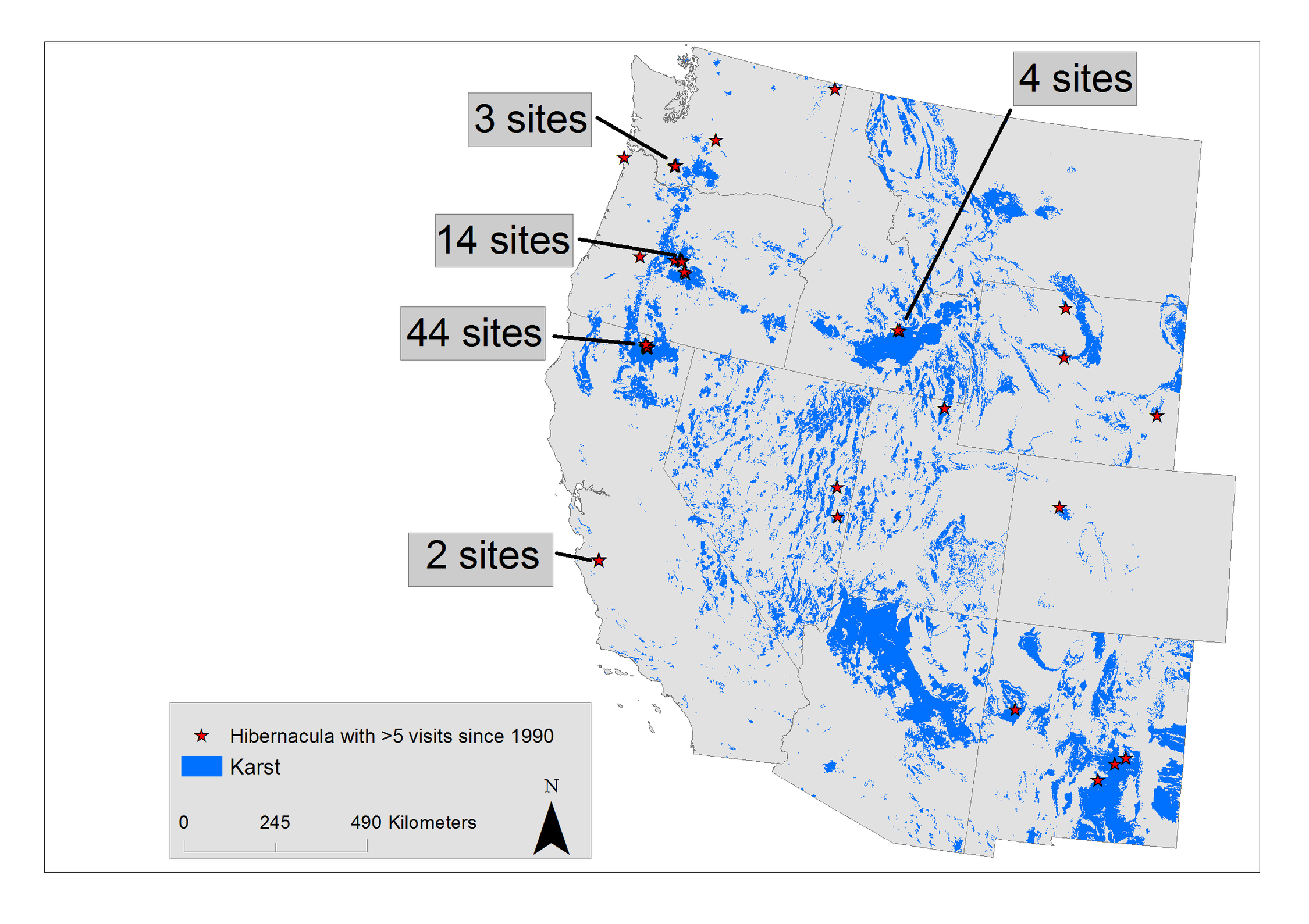

Supplement: S4 Fig — (TIF) [file pone.0205647.s004.tif]

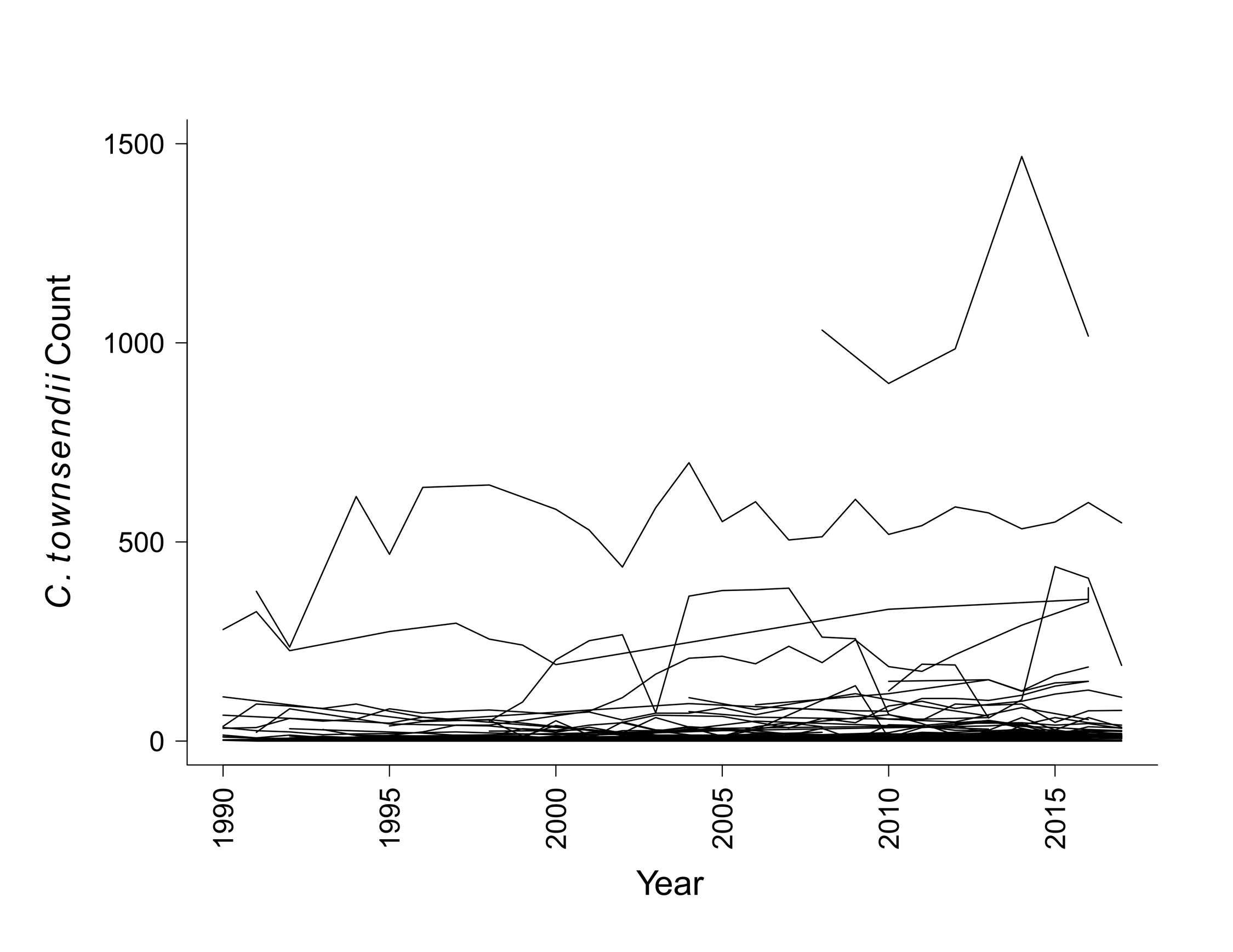

Supplement: S5 Fig — (TIF) [file pone.0205647.s005.tif]

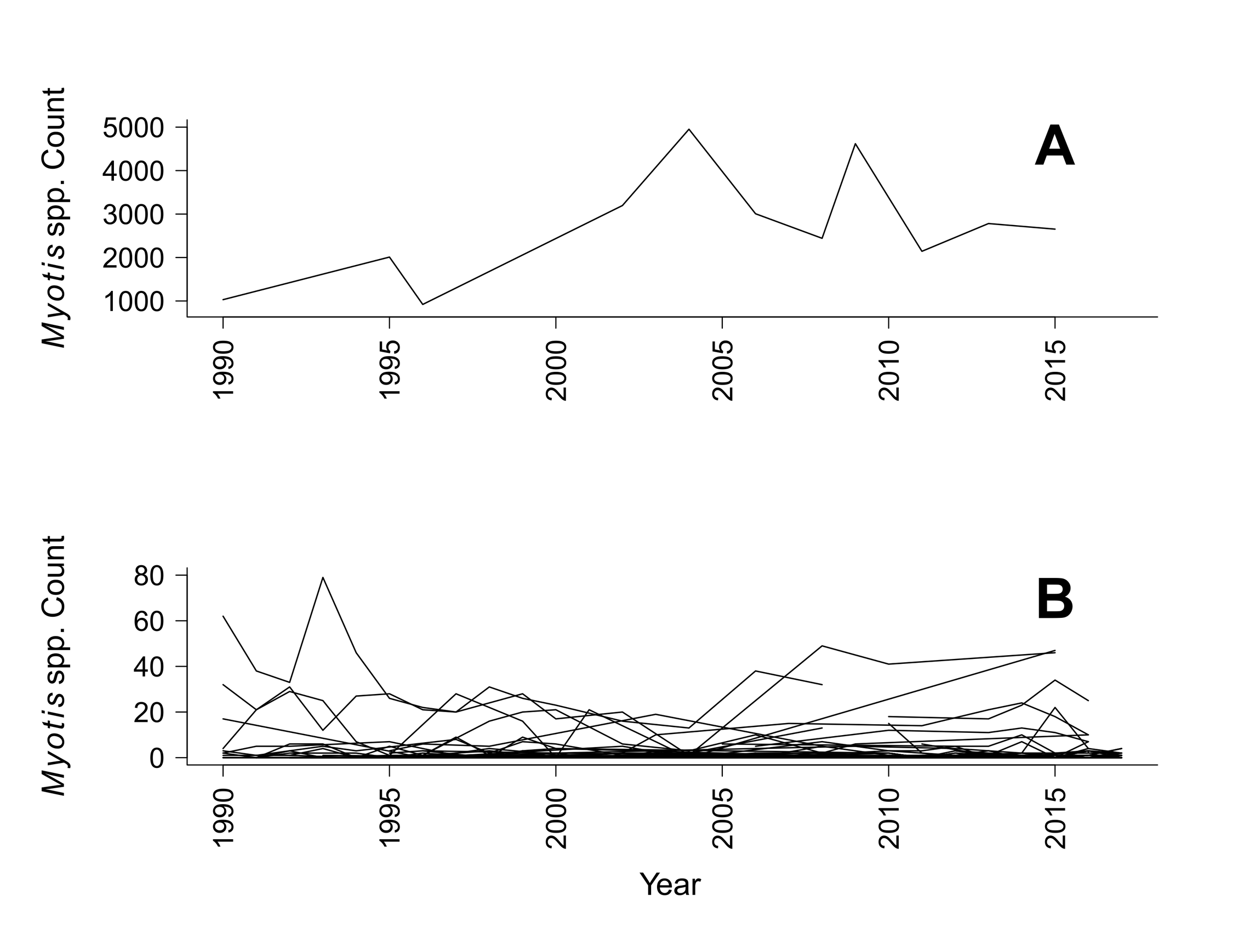

Supplement: S6 Fig — Trends from a single hibernacula in New Mexico are plotted in sub-panel A while the remaining 81 structures are plotted in sub-panel B. (TIF) [file pone.0205647.s006.tif]
