# Supplementary material for: A review of bat hibernacula across the western United States: Implications for white-nose syndrome surveillance and management
Source: PLoS One. 2018 Oct 31;13(10):e0205647. doi: 10.1371/journal.pone.0205647 (PMC6209190; doi:10.1371/journal.pone.0205647)
Supplement: S1 Text — Descriptions of survey efforts by state. (DOCX) [file pone.0205647.s007.docx]

**Supplemental Information**

Descriptions of Survey Efforts by State

**Arizona**

The Arizona Game and Fish Department has had an active bat program with at least one full-time bat biologist since 1992 and has conducted or contracted thousands of roost surveys over the past 25 years. Surveys have been conducted in all seasons, but most efforts have been biased toward the summer maternity season. With the emergence of white-nose syndrome as a threat to hibernating bats, more resources have been directed towards obtaining information on winter bat ecology. Records of winter bat roosts included for Arizona were obtained primarily from surveys conducted during winter months of 2013 and 2014 as part of the “Western Coordinated Multi-State Response to a Deadly, Emerging Threat: White-nose Syndrome in Bats,” a Competitive State Wildlife Grant project undertaken to collect baseline data on bat activity during the winter and to locate and document sites occupied as winter bat hibernacula. Winter roost surveys in 2013 and 2014 included high elevation caves (> 1370m) and select abandoned mines in eastern, southeastern, and north central Arizona to maximize the potential to find hibernating bats. Surveys were conducted by Bat Conservation International and MineGates Environmental, Inc. Other records originate from the Arizona Game and Fish Department’s database and local researchers.

**California**

Records of winter bat roosts in California originate from a wide-variety of sources including from a California Department of Fish and Wildlife database, local researchers, National Parks, and the assembled trip reports from caving grottos. We only included results from cave trip reports when bats were noted as present because these trips were not focused on documenting presence or absence of bats and low numbers of bats could easily have been missed. A statewide survey of *C. townsendii* has been underway since 2014 at known roosts and potential habitat for bats. Related work on *C. townsendii* maternity roosts and hibernacula in the White and Inyo Mountains of eastern California is monitoring inter-roost movement and fidelity using bats marked with PIT tags, as well as conducting repeat winter counts at known hibernacula. Pinnacles National Park has conducted annual counts at two caves where *C. townsendii* overwinter, each of which has registered counts of >100 individuals; *Myotis* spp. have not been observed at Pinnacles. Lava Beds National Monument has conducted regular winter surveys for bats in caves since 1991. To date, 238 of the nearly 800 caves in the monument have been surveyed and *C. townsendii* hibernacula are regularly detected including in three separate caves where >100 individuals have been counted. *Myotis* spp. are rarely detected during these surveys. Work currently underway at Lava Beds aims to understand the structural and environmental conditions that distinguishes caves that support hibernacula from those that do not. Important contributors to data compilation from California include: Betsy Bolster, Kristi Cripe, Joseph Szewczak, and Elizabeth Pierson.

**Colorado**

Accessing potential hibernacula in winter is logistically challenging in Colorado due to topography and snowpack. Consequently, historic records of such roosts over the first half of the 20^th^ century were rare. As equipment such as snow machines improved towards the latter half of the century, survey efforts increased. In Colorado, records of winter bat roosts were queried from the Colorado Parks and Wildlife (CPW) Bat and Scientific Collections databases. The Bat database records referenced here are largely tied to the Bats Inactive Mines Project’s abandoned mine surveys which started in 1990 (Navo 2001, Navo and Krabacher 2005, Hayes et al. 2011). A smaller subset of records from both databases were collected at caves by CPW, the Colorado Natural Heritage Program (CNHP), the US Forest Service (USFS), and local researchers or gleaned from cave grotto trip reports. Records predating 1994, when collections permits were initiated in the state, have been compiled in Armstrong et al. (1994). Survey efforts at caves and mines containing large hibernacula for Colorado (>20 bats) have varied from once every three years where management plans exists, to opportunistic visits only. Management plans tied to surveys have attempted to attain a balance between reducing disturbance while maintaining population trends. The 2011 National Speleological Society’s Convention (Reames 2011) in Glenwood Springs, CO spurred an increase in efforts to survey caves for winter bat use across the state, particularly in areas with high karst resources such as the White River National Forest. Survey efforts by CPW, CNHP, USFS, and the Bureau of Land Management continue at new and known caves and mines as part of the state’s current WNS Surveillance Plan. Surveys that did not yield bats (absence) were not consistently accounted for in the abandoned mines database until 2001 and for cave surveys until 2008. Consequently, survey totals are biased towards presence data but still reflect the low numbers of individuals typically identified at these sites in Colorado and a strong bias towards *C. townsendii* as the species most often identified. A limited number of winter bat roost records outside of those found in caves and mines have historically been noted from buildings, tunnels, and aqueducts with small numbers of bats present (Armstrong et al. 2011). Since 2000, evidence for bats in Colorado using other winter roosts such as rock crevices has grown. *Eptesicus fuscus* were tracked to rock crevices in boulders on the forest floor and in small cliff faces after making short migrations from lower elevation summer maternity roosts in anthropogenic structures (Neubaum et al. 2006). Similarly, *Myotis lucifugus* were tracked from summer maternity and autumn transition roosts in the Crystal River Valley to rock crevices at high elevations in talus slopes (Neubaum 2018). These studies suggest that where rock crevice resources are available in Colorado bats are using short distance migrations in elevation rather than latitude to reach winter roosts. Winter roosts have yet to be documented for many of bat species occurring in Colorado but cave and mine survey efforts along with telemetry studies suggest that rock crevices are likely to be used and warrant future investigation. Important contributors to data compilation from Colorado include: Lea R. Bonewell, Mark A. Hayes, Tom Ingersoll, Nancy LaMantia-Olson, Antoinette J. Piaggio and Elijah Wostl.

**Idaho**

Idaho has a long history of bat work with historic occurrence data for bats dating back to 1895. The earliest known record for a hibernaculum in the state was reported in 1932. Contemporary survey efforts to advance our understanding of bats in Idaho span the past 25 years, initiated in late 1993 by the Idaho Conservation Effort, an interagency conservation program that aimed to remove threats and develop conservation agreements for species at risk of being listed as threatened or endangered under the Endangered Species Act of 1973, as amended (16 USC 1531 et seq.). The primary agencies involved at the time were the US Fish and Wildlife Service (FWS), Bureau of Land Management (BLM), US Forest Service (FS) Northern and Intermountain Regions, Idaho Department of Fish and Game (IDFG), and Idaho Department of Parks and Recreation. Although limited studies and surveys had been conducted in some areas of Idaho prior to 1993, relatively little was known at the time regarding the distribution and status of most bat species in the state. Consequently, during the summer of 1995, and continuing into 1996, the BLM, FS, and IDFG began an intensive effort to determine the status, distribution, and habitat preferences of Idaho’s bat species. During the winters of 1993 and 1995, hibernacula surveys were conducted at several sites that had been previously surveyed by Wackenhut (1990). Data were summarized and sent to the Idaho Conservation Data Center for inclusion in its database (now the Idaho Department of Fish and Game, Idaho Fish and Wildlife Information System, Species Diversity Database). Since 1993, new hibernacula sites have been identified and many placed on a 2-year survey schedule. The Idaho Conservation Effort no longer exists as a formal entity but a strong collaboration exists within the bat community in Idaho to conserve bats. In support of the current project, we queried the Idaho Department of Fish and Game’s Species Diversity Database for hibernacula data. Agencies and entities that contributed hibernacula data to this effort include the Bureau of Land Management, Craters of the Moon National Monument and Preserve, Veolia, Brigham Young University—Idaho, Idaho State University, Idaho Cave Survey, Idaho Department of Fish and Game, US Army Corps of Engineers, US Fish and Wildlife Service, Silver Sage Grotto, US Forest Service Intermountain and Northern Regions, and Northern Rocky Mountain Grotto. The Idaho Department of Fish and Game continues to work with partnering agencies to conduct hibernacula surveys and to conduct white-nose syndrome surveillance, funded in part by the US Fish and Wildlife Service’s Endangered Species Conservation—Recovery Implementation Funds. Finally, although Idaho has advanced its understanding of where some bat species hibernate in the state, the challenge remains to discover where the rest of the bat species winter. Important contributors of bat data from Idaho include: Becky Abel, Idaho Department of Fish and Game; Bryan Bybee, Veolia; Russell Davis, US Army Corps of Engineers; Bill Doering, Veolia; Scott and April Earl, Idaho Cave Survey; Devin Englestead, Bureau of Land Management; Justin Frye, Bureau of Land Management; Katie Gillies, Idaho State University; Daryl Greaser, CaverPilot Consulting; Devon Green, USDA Forest Service, Caribou–Targhee National Forest; David Kampwerth, US Fish and Wildlife Service; Brad Lengas, Utah State University ; Lyle Lewis, Bureau of Land Management; Joel Sauder, Idaho Department of Fish and Game; Silver Sage Grotto; Todd Stefanic, National Park Service, Craters of the Moon National Monument and Preserve; Martha Wackenhut, Idaho Department of Fish and Game; Jericho Whiting, Brigham Young University–Idaho; Ross Winton, Idaho Department of Fish and Game; and Gary Wright, Bureau of Land Management.

**Nevada**

Nevada has had an active program studying bats and their use of abandoned mines and caves for approximately 20 years. To date, some 5,000 mines and >100 caves have been assessed for bat use. The program has increased in intensity in the last decade, and currently approximately 350 abandoned mines are surveyed annually in order to include wildlife friendly recommendations to secure the mines. Current research focuses on: 1) describing variation in roost climate conditions; 2) locating and describing hibernacula; 3) understanding the different types of roost use in mines and caves; and, 4) understanding bats’ acceptance of different types of bat gate designs. Regarding hibernacula investigations, cavernicolous roosts are surveyed year round and assessed for hibernacula potential based on two variables that have proven to be very important to hibernating bats in Nevada: presence of airflow and/or presence of vertical features that may serve as cold-air sinks. Sites deemed as potential hibernacula are resurveyed during winter to understand hibernation use. Data collected on the physical characteristics, bat use, and climate of hibernacula are used to refine search criteria when identifying additional potential hibernacula. Since well over 300,000 abandoned mine features exist in Nevada, understanding the variation in hibernacula use of abandoned mines is expected to take some time.

**New Mexico**

Winter bat research in New Mexico has been documented by cavers and agencies since at least the 1960s. Though some large summer roosts are on private land, most documented winter roosts are on federal land. In general, attention has been directed toward large roosts in New Mexico. Roosts that occasionally have only a few bats at random times of year are not regularly documented. The Bureau of Land Management in New Mexico has conducted hibernacula counts going back to at least 1967. The Lincoln National Forest, Guadalupe District has put new effort into documenting their bats in the last 5 years. El Malpais National Monument has recently started a project to document bat use >300 lava tubes. Many mines have also been surveyed and protected for bat use in New Mexico but it has been difficult to obtain that information. Laura Baumann, National Park Service, helped compile the data from El Malpais National Monument.

**Montana**

Use of caves and mines as hibernacula by bats has been known in Montana since at least the 1930’s by the general public, biologists, and recreational cavers (Campbell 1978, Hendricks 2012), however most of these early observations were incidental and structured efforts focused on single caves or caves within a single mountain range or area (e.g. Worthington 1991, Hendricks 2000). In 2011 state and federal agencies began working with local cavers to begin a comprehensive survey of the state’s caves to establish baseline counts and document the community of species overwintering in caves. Reports from cavers have been used to prioritize survey of caves that appear to be used by bats, with biologists attempting to access these during winter and conduct formal surveys. In 2016 the state, in partnership with federal and tribal agencies, began a WNS surveillance program which has contributed additional annual count data for five caves with significant aggregations of hibernating bats. To date, over 110 of the 411 known caves have been formally surveyed as part of these efforts. Data from these surveys is likely biased toward bat use as these surveys targeted caves where bats were previously observed, significant guano depositions had been found, or cave attributes such as length, depth, temperature, and humidity suggest suitability for winter use. Although hundreds of caves and thousands of mines remain unsurveyed, the data provided by this project and previous survey efforts indicates that few caves or mines are significant hibernacula, and most caves are used by few individuals, if at all. However, counts at several caves in the central and eastern regions of the state have winter counts that exceed 200 individuals and one cave exceeds 1,500 *Myotis* spp. individuals. Of the 15 species present in Montana, eight have been observed during winter surveys of caves and mines including Townsend’s big-eared bat (*Corynorhinus townsendii*), big brown bat (*Eptesicus fuscus*), little brown myotis (*Myotis lucifugus*), long-eared myotis (*M. evotis*), fringed myotis (*M. thysanodes*), long-legged myotis (*M. volans*), northern myotis (*M. septentrionalis*), and western small-footed myotis (*M. ciliolabrum*). Evidence from acoustic surveys conducted in the winter increasingly supports the hypothesis that rock outcrops and talus slopes may be important overwintering habitat for many of the state’s resident species (D. Bachen pers. comm). Data from this project and previous efforts have been archived in databases at the Montana Natural Heritage Program. Important contributors to data compilation from Montana include: Hans Bodenhamer and students with the Big Fork Highschool Cave Club; Lauri Hanauska-Brown, Montana Fish, Wildlife, and Parks; Paul Hendricks, Montana Natural Heritage Program; Members of the Northern Rocky Mountain Grotto; Ellen Whittle, Montana Natural Heritage Program;

**Oregon**

Previous efforts to compile historic records of winter bat roost surveys in Oregon (The Bat and The Caves Databases; Ormsbee and Risdal 2007, Ormsbee et al. 2010) helped account for the wide-range of years included in the Oregon dataset. These databases were populated with data from reports, publications, personal data sets from research and survey efforts, and museum records across all ownerships and all seasons. Oregon winter roost data were also provided from the USFS Natural Resource Management (NRM) Wildlife data base (<https://www.fs.fed.us/nrm/index.shtml>) and the BLM Geographic Biotic Observations (GeoBob) database (<https://www.blm.gov/policy/im-or-2013-022>). While many of the surveys associated with these data were conducted by BLM and USFS biologists, other collaborators were essential partners for winter surveys and included other state and federal biologists, grotto members, private land owners, and volunteers from local communities. For example, The Deschutes National Forest, Prineville BLM, and Lakeview BLM have collaborated with local grotto members, Oregon Department of Fish and Wildlife, and community volunteers to conduct winter bat surveys of caves in central Oregon since 1984. Thirty-three caves have had 1 or more surveys in that time and 13 have been surveyed regularly across the years. Personnel from the USFS and BLM Abandoned Mines Programs also have collaborated with biologists to identify potential winter bat roost habitat and conduct surveys in order to prescribe appropriate closure methods for winter hibernacula. This effort has involved review of several hundred sites by USFS and BLM mine specialists and biologists followed by surveys to determine bat use. Surveys have been conducted by agency personnel and contractors such as Bat Conservation International. In 2012, an interagency team (USFS, BLM, USFWS, NPS, and Oregon and Washington State Wildlife Departments) compiled data on known and potential winter bat roost habitat as part of a risk assessment for WNS. These data also were provided for this paper.

**Utah**

Assessing potential bat hibernacula in Utah is challenging due to inaccessibility and snow cover during winter. Some of the Utah bat hibernacula reported here required several kilometers of travel on skis or snowshoes. As a result, a number of possible Utah bat hibernacula have not been examined because no way has yet been found to reach them in winter. There are a few natural caves in Utah, though very few of them are solution caves. However, there are many abandoned mines, approximately 7,000 of which have been closed. The number of still-open abandoned mines in Utah is not known with certainly, but is probably >10,000. Despite the existence of natural caves and a great number of abandoned mines in Utah, relatively few of the known caves or mines are thought to provide conditions suitable for bat hibernation (Twente 1960). Sources used to compile Utah bat hibernacula data included bat databases assembled by the Utah Division of Wildlife Resources and the Utah Division of Oil, Gas and Mining. The Utah Division of Oil, Gas and Mining’s Abandoned Mine Reclamation Program has conducted warm season and cold season surveys of bats in abandoned mines in Utah since 1993, and the Utah Division of Wildlife Resources’ Wildlife Conservation Program has collected bat data in Utah for more than 20 years. These sources have been supplemented by data from other sources, especially recent surveys by Timpanogos Cave National Monument staff of caves along the lower Provo River in the vicinity of the Monument. Utah Division of Oil, Gas, and Mining allowed their data from mines to be shared.

**Washington**

Data from winter bat surveys in Washington have come from a variety of sources and represent decades of work. The surveys have involved coordination and collaboration between multiple state and federal agencies, organizations, and private landowners and companies. For example some of the Washington records came from the USFS Natural Resource Management (NRM) Wildlife data base (<https://www.fs.fed.us/nrm/index.shtml>) and the BLM Geographic Biotic Observations (GeoBob) database (<https://www.blm.gov/policy/im-or-2013-022>). Some of the earliest documented winter bat surveys, beginning in 1965, were at caves in Skagit, Skamania and Klickitat counties (Senger et al. 1974). These surveys documented hibernacula for multiple species, including Townsend’s big-eared bat (*Corynorhinus townsendii*), long-legged myotis (*Myotis volans*), western long-eared myotis (*M. evotis*), and little brown bat (*M. lucifugus*). Species identification were based on visual observations; consequently, because some *Myotis spp*. are not reliably identified based on visual observation, some of the records may not be accurate to species. Hibernacula surveys continued into the 1980s at caves in Skamania and Klickitat counties as well as at abandoned mines in Pend Oreille, Stevens and Okanagan counties (Perkins et al. 1990). These hibernacula surveys primarily documented Townsend’s big-eared bat, but various *Myotis* spp. were also documented in smaller numbers (*n* = 1–7). During this same time period, the Gifford Pinchot, Wenatchee, Okanogan, and Colville National Forests along with Washington Department of Fish and Wildlife (WDFW) began conducting hibernacula surveys at >25 caves and abandoned mines. Some of these hibernacula have been consistently monitored for years for Townsend’s big-eared bats. Washington Department of Natural Resources also conducted hibernacula counts at caves in Skagit County in the 1980s as well as in the late 2000s. These caves were mainly used by Townsend’s big-eared bat. The Bureau of Land Management (BLM) also conducted hibernacula counts at abandoned mines in Ferry, Stevens, and Pend Oreille counties in the 1990s. More recently, the BLM contracted with Bat Conservation International for abandoned mine assessments, several of which occurred during winter. In the mid-2000s, Seattle City Light investigated winter bat use at caves and abandoned mines in Pend Oreille County. The Department of Defense conducted hibernacula counts at a cave in Douglas County used by >200 Townsend’s big-eared bats. In Thurston, Skagit and Island counties, private citizens along with state partners (e.g., Washington State Parks) conducted winter surveys and found California myotis (*M. californicus*), Townsend’s big-eared bat, big brown bat (*Eptesicus fuscus*), silver-haired bat (*Lasionycteris noctivagans*), and either little brown bat or Yuma myotis (*M. yumanensis*) roosting in buildings, under bridges and in a cave. After the discovery of white-nose syndrome (WNS) in Washington in 2016, WDFW and partners have been assessing winter bat records and using acoustic detectors to identify new winter roosts where WNS surveillance and population monitoring can occur.

**Wyoming**

Winter bat surveys have been conducted in Wyoming since 1992, including periodic monitoring at select known hibernacula as well as prospecting for previously unrecorded hibernacula in known cave and mine structures. Surveys are conducted using a standardized protocol (Hester and Grenier 2005), with most surveys conducted by Wyoming Game and Fish Department personnel. All surveys are recorded in a Wyoming Game and Fish Department database, including surveys where no bats were detected. All cave and mine structures surveyed during the hibernation season were included as all visits were for the express purpose of conducting bat surveys. Over 600 cave and abandoned mine structures have been identified in the state; approximately 200 of these have been surveyed during the hibernation season. Myotis are often found hibernating with *C. townsendii*, with *Myotis* spp. generally roosting interior to *C. townsendii*. Environmental conditions in several *Myotis* spp. hibernacula have been monitored continuously over several years and were found to be within the growth range for *P. destructans* (Beard 2015). Important contributors to data compilation from Wyoming include: Martin Grenier, Ducks Unlimited; and Bob Luce, Wyoming Game and Fish Department, retired.

**Supplemental Literature Cited**

Armstrong, D. M., J. P. Fitzgerald, and C. A. Meaney. 2011. Mammals of Colorado, 2^nd^ ed. University of Colorado Press, Boulder, Colorado, USA.

Beard, L. 2015. Surveillance of hibernating bats and environmental conditions at caves and abandoned mines in Wyoming. Pages 163-193 *in* Threatened, Endangered, and Nongame Bird and Mammal Investigations (A. C. Orabona and C.K. Rudd, Editors). Wyoming Game and Fish Department Nongame Program, Lander, WY, USA.

Campbell, N.P. 1978. Caves of Montana. Montana College of Mineral Science and Technology.169 p.

Hayes, M.A., Schorr, R.A., Navo, K.W., 2011. Hibernacula selection by Townsend's Big-Eared Bat in Southwestern Colorado. Journal of Wildlife Management 75, 137-143.

Hendricks, P. 2012. Winter records of bats in Montana. Northwestern Naturalist 93, 154-162.

Hendricks, P., Genter, D.L., Martinez, S. 2000. Bats of the Azure Cave and the Little Rocky Mountains, Montana. Canadian Field Naturalist 114, 89-97.

Hester S. G., and M. B. Grenier. 2005. A Conservation Plan for Bats in Wyoming. Wyoming Game and Fish Department, Lander, Wyoming, USA.

Navo, K. W. 2001. The survey and evaluation of abandoned mines for bat roosts in the West: guidelines for natural resource managers. Proceedings of the Denver Museum of Nature and Science Series 4:1-12.

Navo, K.W., and P. Krabacher. 2005. The use of bat gates at abandoned mines in Colorado. Bat Research News 46, 1-8.

Neubaum, D.J. 2018. Unsuspected retreats: autumn roosts and presumed hibernacula used by little brown myotis in Colorado. Journal of Mammalogy: *in revision*.

Neubaum, D.J., O'Shea, T.J., Wilson, K.R., 2006. Autumn migration and selection of rock crevices as hibernacula by big brown bats in Colorado. Journal of Mammalogy 87, 470-479.

Ormsbee P.C. and L. Risdal. 2007. The Caves Database: a compilation of cave sites with bat detections and related attributes spanning 1883 to 2010 collected in the Pacific Northwest. USDA Forest Service Region 6.

Ormsbee P.C., L. Risdal, and A. H. Hart. 2010. The Bat Database: a compilation of bat species detections and related attributes spanning 1883 to 2010 collected in the Pacific Northwest. USDA Forest Service Region 6.

Perkins, J.M., Barss, J.M., Peterson, J., 1990. Winter records of bats in Oregon and Washington. Northwestern Naturalist 71, 59-62.

Reames, S. 2011. Caves and Karst of NW Colorado: a guidebook for the 2011 convention of the National Speleological Society. National Speleological Society, Huntsville, Alabama, USA.

Senger, C.M., Senger, R., Senger, D., Senger S. 1974. Winter records of myotid bats in western Washington. Murrelet 55, 13-14.

Twente, J.W., 1960. Environmental problems involving the hiberation *[sic]* of bats in Utah. Proceedings of the Utah Academy of Sciences, Arts, and Letters 37, 67-71.

Worthington, D. J. 1991. Abundance and distribution of bats in the Pryor Mountains of south central Montana and north eastern Wyoming. Montana Natural Heritage Program Report. 23 p. https://archive.org/details/1991marchabundancedistribwortrich
